# Comparative Outcomes of Ultrasound-Guided Versus Fluoroscopy-Guided Percutaneous Transhepatic Biliary Drainage (PTBD) Due to Malignant Obstructive Jaundice: A Single-Center Retrospective Analysis

**DOI:** 10.3390/life16101759

**Published:** 2026-10-20

**Authors:** Marco Giurdanella, Domenico Laganà, Caterina Battaglia, Giuliana Conti, Nicola De Rosi, Francesco Manti, Giuseppe Mazzeo, Marco Fulciniti, Pier Paolo Arcuri, Claudio Maglia, Francesco Tiralongo, Roberto Minici

**Affiliations:** 1 Radiology Unit, Dulbecco University Hospital, 88100 Catanzaro, Italy; marco-giurdanella@virgilio.it (M.G.); caterina.battaglia@unicz.it (C.B.); giuliana.conti92@gmail.com (G.C.); derosinicola@gmail.com (N.D.R.); manti@unicz.it (F.M.); giuseppemazzeo94@gmail.com (G.M.); marco.fulciniti@aourenatodulbecco.it (M.F.); arppaolo@alice.it (P.P.A.); claudiomaglia@hotmail.it (C.M.); 2 Department of Experimental and Clinical Medicine, Magna Graecia University of Catanzaro, 88100 Catanzaro, Italy; domenico.lagana@unicz.it; 3 Radiology Unit 1, Department of Medical Surgical Sciences and Advanced Technologies “GF Ingrassia”, University Hospital Policlinico “G. Rodolico-San Marco”, University of Catania, 95123 Catania, Italy; tiralongofrancesco91@hotmail.it

**Keywords:** biliary drainage, fluoroscopy, ultrasound, complications, radiation exposure, percutaneous, PTBD, transhepatic, PTC, jaundice

## Abstract

**Background**: Percutaneous transhepatic biliary drainage (PTBD) remains a cornerstone in managing obstructive jaundice, particularly when endoscopic retrograde cholangiopancreatography (ERCP) is unsuccessful or contraindicated. This study aims to compare ultrasound-guided (US-PTBD) and fluoroscopy-guided (F-PTBD) techniques regarding procedural time, radiation exposure, technical success, and complication rates. **Methods**: This retrospective, single-center study included patients with malignant obstructive jaundice who underwent PTBD between January 2018 and December 2025. Patients were stratified based on the imaging modality used for the bile duct puncture (US-PTBD vs. F-PTBD). Procedural characteristics, including dose–area product (DAP), fluoroscopy time, complication rates, and technical success, were analyzed using statistical methods appropriate for continuous and categorical data. **Results**: A total of 133 patients (163 PTBD procedures) were included. US-PTBD demonstrated significantly lower DAP (4.1 ± 1.9 Gy·cm^2^ vs. 6.2 ± 1.5 Gy·cm^2^; *p* < 0.001) and shorter procedure time (42 ± 7 min vs. 56.6 ± 6 min; *p* < 0.001) compared to F-PTBD. Both techniques had comparable technical success rates (91.1% vs. 82.2%; *p* = 0.158). Complications occurred in 10.2% of patients in the F-PTBD group and 6.7% in the US-PTBD group, with no statistically significant difference between groups (*p* = 0.489). Major complications, including bleeding and cholangitis, occurred exclusively in the F-PTBD cohort. US-PTBD resulted in fewer Glisson capsule punctures, suggesting reduced procedural trauma. **Conclusions**: Although a significant access side imbalance was present between cohorts, US-PTBD offers distinct advantages over F-PTBD, including reduced radiation exposure, shorter procedure times, and a comparable safety profile. These findings highlight the potential of US guidance as a safer alternative for PTBD, particularly in patients with coagulopathy or complex anatomy. Further multicenter studies with larger populations are warranted to confirm these benefits and refine patient selection criteria.

## 1. Introduction

Percutaneous transhepatic biliary drainage (PTBD) remains a cornerstone in managing obstructive jaundice, a complex condition that poses significant diagnostic and therapeutic challenges. Jaundice, with its multifactorial etiologies ranging from malignant cholangiocarcinoma to benign biliary strictures, underscores the pivotal role of precision in diagnosis and treatment [1,2]. Historically, PTBD evolved from percutaneous transhepatic cholangiography (PTC) to become an essential component of interventional radiology, thus allowing interventionists to offer both diagnostic and therapeutic procedures. With its ability to provide direct access to the biliary system, PTBD is often employed when endoscopic retrograde cholangiopancreatography (ERCP) is unsuccessful or contraindicated due to altered anatomy such as postsurgical biliodigestive anastomoses, or complex difficult-to-cross or hilar biliary strictures [3,4]. The indications for PTBD span a wide range, from malignant obstructions due to cholangiocarcinoma, pancreatic cancer, ampullary malignancies and metastatic disease, to non-malignant conditions such as primary sclerosing cholangitis, iatrogenic strictures and biliary atresia [5]. Advances in radiological imaging, including computed tomography (CT), magnetic resonance cholangiopancreatography (MRCP), and endoscopic ultrasound (EUS), have significantly refined the diagnosis and staging of biliary disease, allowing for tailored PTBD procedures based on individual patient anatomy and disease state [6,7]. In particular, MRCP has proven useful in delineating biliary anatomy without radiation exposure, complementing PTBD’s capabilities [8].

Mitigating the complications inherent to percutaneous transhepatic biliary drainage (PTBD) plays a pivotal role in enhancing overall patient outcomes. Turan et al. ([9]) evaluated the incidence of complications and associated risk factors following PTBD performed after failed ERCP. The study included a total of 331 patients who underwent PTBD across five centers in the Netherlands. The overall 30-day complication rate was 62.8%. Non-infectious complications were observed in 34.7% of patients, while infectious complications occurred in 40.6% of patients. Specific infectious complications included cholangitis (26.3%), sepsis (24.6%), abscess formation (2.7%), and cholecystitis (1.3%). The 30-day all-cause mortality rate was 17.2%, with 27 deaths (8.2%) appearing to be a direct or indirect result of the PTBD procedure. A re-intervention was performed within 30 days after the initial PTBD in 73.7% of 224 patients. Risk factors for infectious complications included internal drainage (OR 2.12) and drain obstruction (OR 2.60). The number of re-interventions within 30 days was a significant risk factor for non-infectious complications (OR 1.52) [9]. Nonetheless, the imaging guidance modality used during PTBD procedures can significantly affect complication rates and should not be overlooked. Both fluoroscopy-guided and ultrasound-guided percutaneous transhepatic biliary drainage (F-PTBD and US-PTBD, respectively) procedures are available, with each technique presenting unique benefits and drawbacks. Importantly, PTBD is frequently performed as a hybrid workflow: even when ultrasound is used for duct puncture, fluoroscopy is commonly employed for cholangiography, guidewire advancement, and catheter/stent positioning. Therefore, the clinically meaningful comparison between ‘US-guided’ and ‘fluoroscopy-guided’ approaches often lies in the access step (biliary duct puncture), which can influence the number of capsule passes, fluoroscopy time, and overall radiation exposure [10,11]. F-PTBD, the conventional approach, boasts a well-defined and standardised protocol. However, it is not without risks; complication rates reported across series vary widely depending on case-mix and complication definitions, and major complications—particularly bleeding and infectious events—remain clinically relevant [12,13,14,15]. These complications can encompass bleeding, pancreatitis, bile leaks, and infections, underscoring the inherent challenges of traversing the intricate biliary anatomy [12,13,14]. The advent of US-PTBD has introduced the potential for reduced complication rates, particularly in terms of hepatic capsule puncture frequency and reduced radiation exposure, as measured by Dose Area Product (DAP) [16,17,18]. This reduction aligns with growing efforts to mitigate radiation risks, especially given cumulative exposure concerns for both patients and healthcare providers [19]. While ultrasound guidance has become the gold standard in many experienced centers, particularly in Asian countries where it is widely adopted for puncture, fluoroscopy-guided puncture remains in use in various institutions worldwide, often due to operator preference or resource availability [20,21]. Furthermore, while the theoretical safety of ultrasound is established, there is a need to strictly quantify the reduction in radiation exposure (Dose Area Product—DAP) and procedural time to reinforce the adherence to ALARA (As Low As Reasonably Achievable) principles in interventional radiology [22]. However, current literature provides limited evidence on the direct comparison between ultrasound-guided and fluoroscopy-guided PTBD, particularly regarding their safety profiles and cumulative radiation exposure to patients and operators, underscoring the need for further investigation to guide clinical decision-making and institutional protocol changes.

This retrospective analysis was designed to compare two image-guidance strategies used in real-world PTBD practice—ultrasound-guided versus fluoroscopy-guided initial duct puncture—in patients with malignant obstructive jaundice, evaluating procedural time, radiation exposure, technical success, and complications, and providing side-specific (right vs. left) analyses to address an area where comparative data remain limited.

## 2. Materials and Methods

### 2.1. Study Design

This study was designed as a retrospective single-center observational cohort study based on data collected from patients who underwent PTBD for obstructive jaundice from January 2018 to December 2025. The inclusion criteria, met by all patients, were as follows: (I) diagnosis of obstructive jaundice due to malignancies; (II) prior failed ERCP (Endoscopic Retrograde Cholangiopancreatography) or ERCP not feasible due to anatomical or clinical reasons; (III) evaluation by a multidisciplinary team consisting of oncologists, surgeons, anesthesiologists, endoscopists, and interventional radiologists.

The exclusion criteria were: (I) multiple metastases that prevent the placement of biliary drainage through healthy parenchyma; (II) abundant ascitic effusion; (III) compatible preprocedural coagulation testing, according to Society of Interventional Radiology consensus guidelines [23]. Patients previously treated with EUS-guided biliary drainage were not present in the study cohort.

The investigation was conducted in accordance with the Declaration of Helsinki. All patients signed written informed consent before undergoing percutaneous treatment. The study protocol was approved by the local Ethics Committee (Protocol No. 209/27 June 2024—Comitato Etico Territoriale Regione Calabria, Italy).

### 2.2. Treatment

In all cases, a preliminary abdominal CT or MRI scan was performed to plan the treatment. PTBD was performed in dedicated angiographic suites by two interventional radiologists with 10 and 30 years of experience, respectively, who consistently alternated between the roles of primary and assistant operator, ensuring an equal distribution of expertise across both study groups. The choice between ultrasound-guided and fluoroscopy-guided initial puncture was based on procedural judgment considering ductal visibility on ultrasound, anatomical features, access side, and operator preference/experience. The patient’s laboratory panel, including liver enzymes and coagulation tests, was preliminarily assessed in accordance with the Society of Interventional Radiology consensus guidelines to minimise the risk of bleeding as much as possible [23]. Percutaneous puncture was performed using the “Neff Percutaneous Access Set” kit (Cook Medical, Bloomington, IN, USA) for both methods.

**Figure 1:**
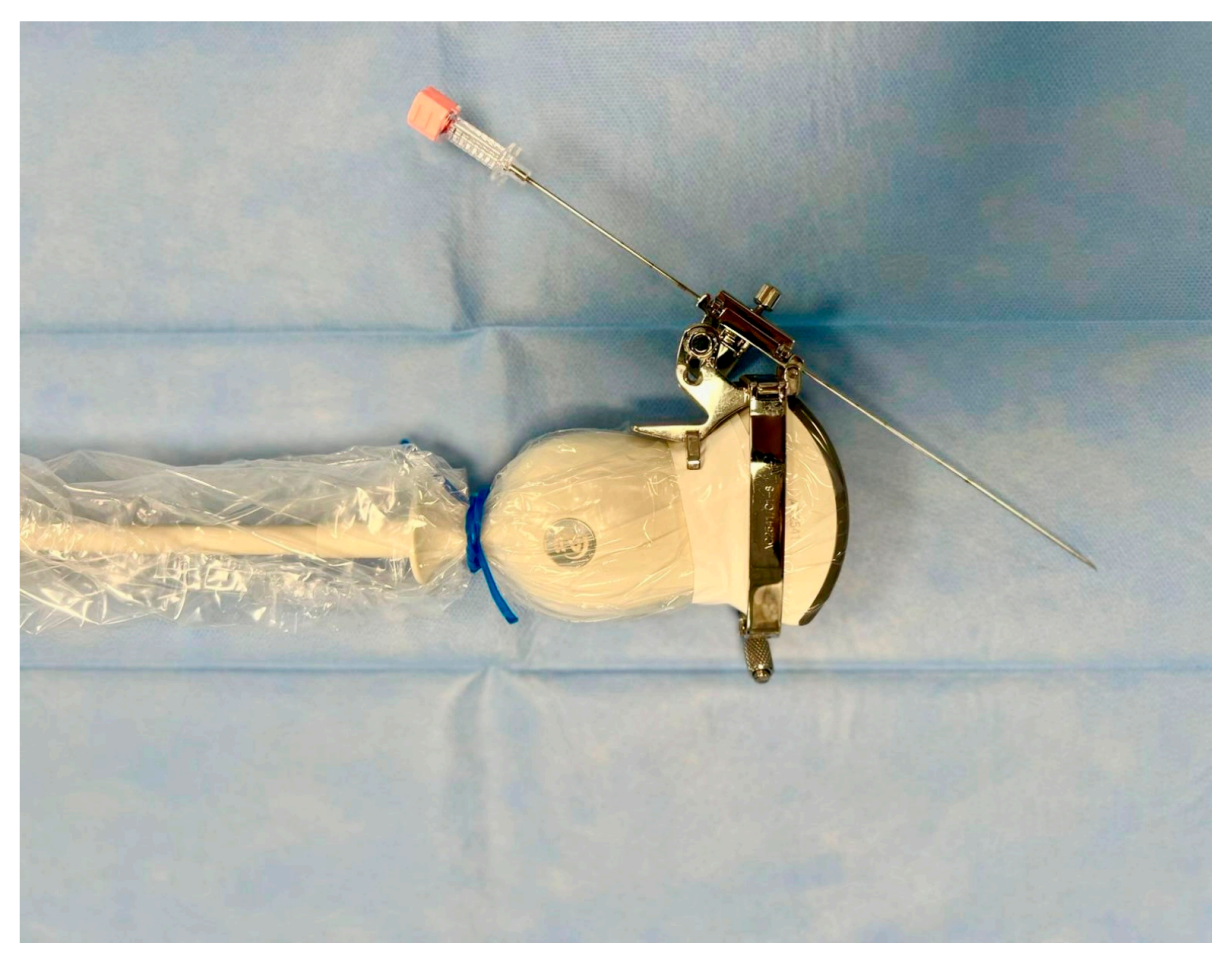
Sterile ultrasound probe for an in-plane puncture with a needle guidance system.

Before every intervention, the patient underwent deep sedation. Procedures were categorized by the imaging modality used for the initial biliary duct puncture: (i) US-PTBD, when puncture was performed under real-time ultrasound guidance, and (ii) F-PTBD, when puncture was performed under fluoroscopy guidance. After duct access, cholangiography, guidewire manipulation, and catheter placement were performed under fluoroscopy in both groups, as per routine practice [10]. In the F-PTBD workflow, the operator targeted a peripheral intrahepatic duct under fluoroscopic guidance based on pre-procedural cross-sectional imaging, advancing the needle toward the expected duct location as previously described by Covey et al. [24]. The US-guided puncture was performed with a sterile 22G Chiba needle mounted laterally to the ultrasound probe within a needle guidance system, as shown in Figure 1.

**Figure 2:**
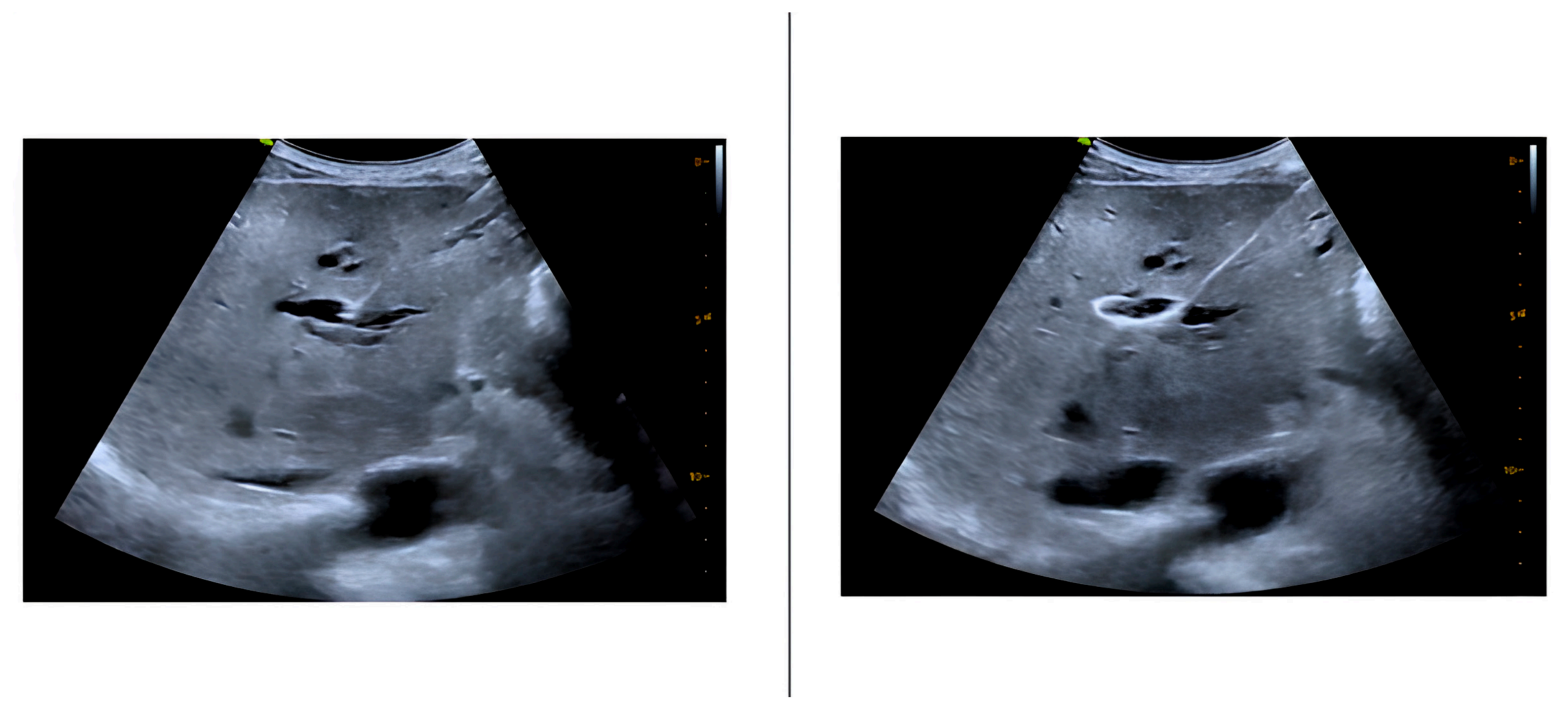
Ultrasound images assessing the puncture of the bile duct and the guidewire insertion.

The ultrasound guidance has made possible the direct visualization of the ductal dilation degree (Figure 2).

**Figure 3:**
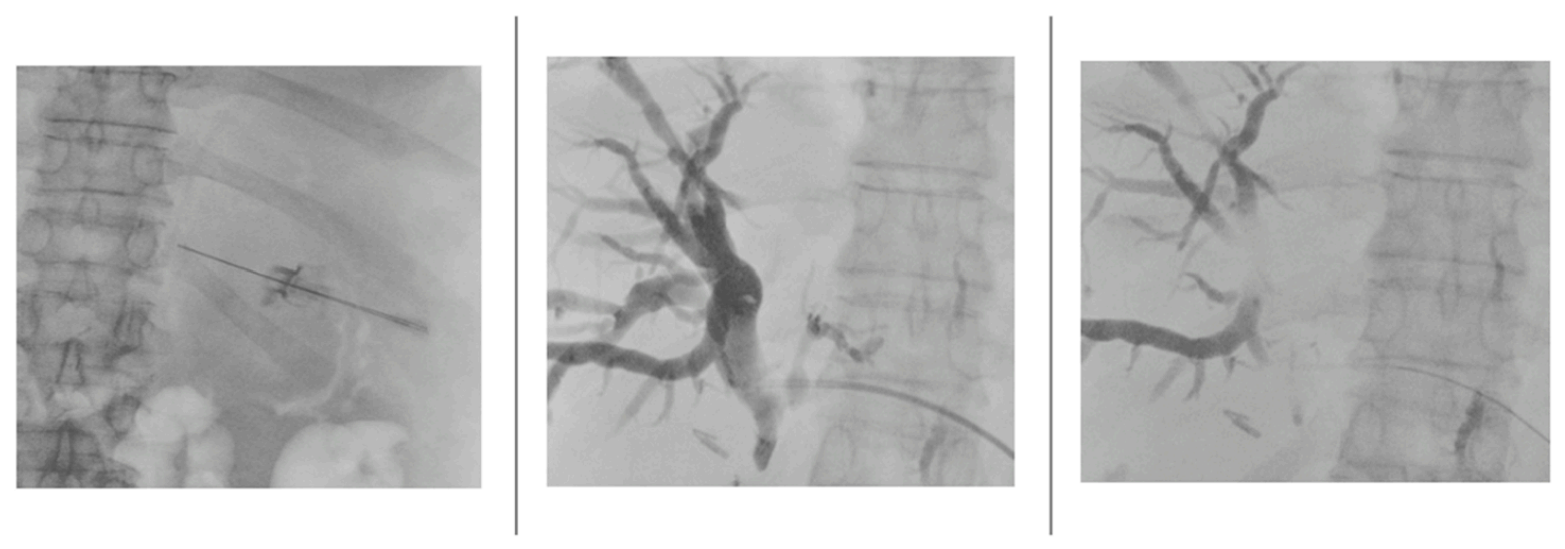
Images highlighting key moments of the cholangiography procedure, from needle insertion and visualization of the bile ducts to opacification and drainage placement.

Once the lumen of the bile duct was penetrated and the needle stylet removed, the needle was disconnected from the needle guidance system to stably inject an iodinated contrast agent (Xenetix 250 (Guerbet, Villepinte, France)) mixed in a 1:1 ratio with saline to opacify the biliary tree under fluoroscopy guidance (Figure 3).

**Figure 4:**
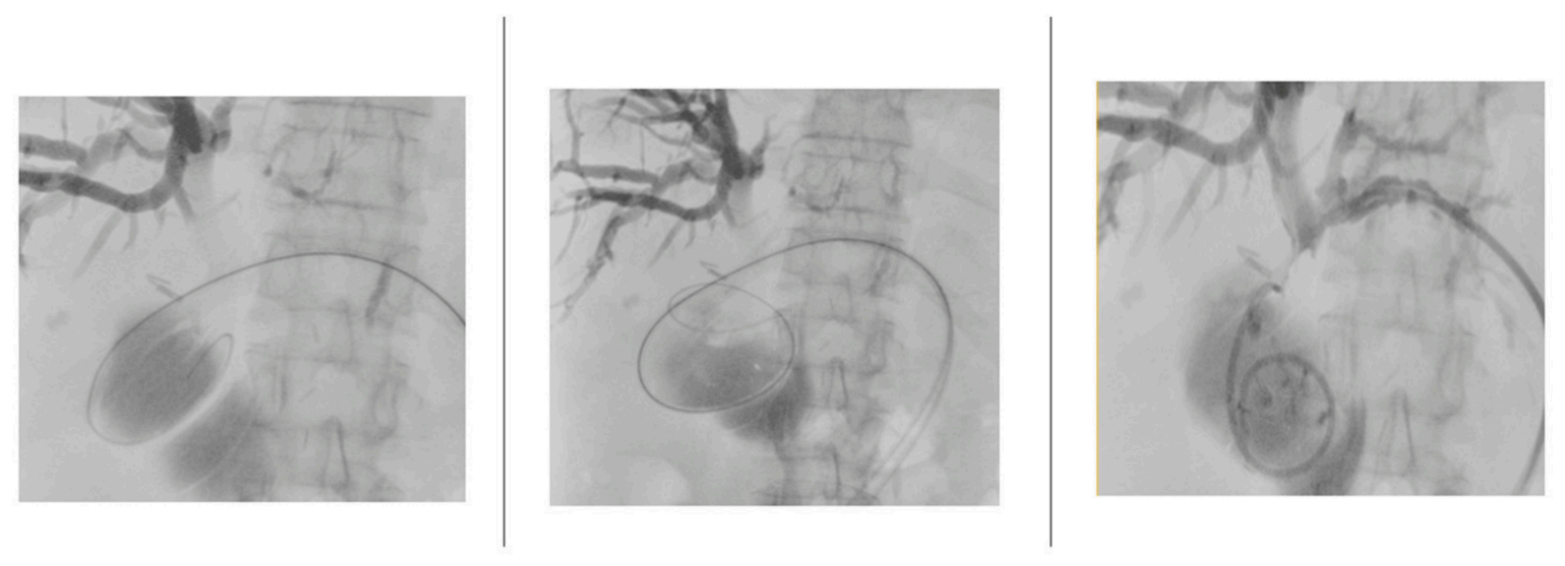
Images depicting the insertion of the guidewire up to the duodenum and the placement of the biliary drainage.

After fluoroscopic visualization of the biliary system, a 0.018” Cope Mandril Nitinol guidewire with platinum tip (Cook Medical) was inserted through the Chiba needle, followed by replacement with an introducer equipped on a tri-axial system to stabilize the access. Subsequently, the 0.018” guidewire was replaced by a standard-type 150 cm angled hydrophilic guidewire (Radifocus, Terumo) to cross the stenosis up to the duodenum. This guidewire was replaced by a 0.035” 145 cm Amplatz-type extra stiff guidewire, over which an 8/10/12 Fr internal–external biliary drainage catheter was positioned (Figure 4).

### 2.3. Definitions

Procedure time was defined as the interval from skin puncture to completion of catheter placement and securement. Fluoroscopy time and dose–area product (DAP, Gy·cm^2^) were recorded automatically by the fluoroscopy unit [25]. Glisson’s capsule punctures were defined as the number of needle passes through the hepatic capsule required to achieve duct access, as documented in the procedural report. Technical success was defined as successful catheter placement within the obstructed biliary system with effective internal–external or external biliary drainage confirmed at the end of the procedure. In addition to technical success, early clinical effectiveness was assessed through total serum bilirubin reduction at 5–7 days after PTBD, expressed both as absolute change and percentage reduction from baseline, when laboratory follow-up was available. Technical failure was defined as inability to access the target biliary tree and/or inability to place a functioning drainage catheter across the intended tract. In cases of technical failure, patients were managed according to multidisciplinary clinical judgment, including repeat percutaneous drainage attempt, alternative access route, or other biliary decompression strategies when feasible. Complications were classified according to the CIRSE classification system [26]. Minor complications (Grades 1–2) included mild pain, nausea, vomiting, and abdominal wall hematoma in the absence of significant bleeding, requiring no treatment or only conservative management. Major complications (Grades 3–6) included clinically significant hemorrhage, post-procedural cholangitis requiring intensive care, or other adverse events requiring secondary procedures, prolonged hospitalization (>48 h), or leading to substantial morbidity.

### 2.4. Statistical Analysis

For categorical variables, absolute and relative percentage frequencies are shown [27]. Continuous data are depicted as mean ± standard deviation [28]. Statistical differences were assessed using an unpaired Student’s *t*-test for continuous normally distributed data, the Chi-squared/Fisher’s exact test for categorical data, and the Mann–Whitney test for continuous data that were not normally distributed, as appropriate [29,30]. To evaluate independent predictors of procedural outcomes and control for potential confounding variables (such as access side and underlying tumor etiology), multivariable regression analyses were performed. A *p*-value < 0.05 was considered statistically significant. Multivariable logistic regression models were constructed for binary outcomes (technical success and post-procedural complications), reporting adjusted odds ratios (aORs) with 95% confidence intervals (95% CIs). Multivariable linear regression models were applied for continuous outcomes (Dose–Area Product [DAP], total procedure time, fluoroscopy time, and number of punctures), reporting regression coefficients (beta) and standard errors (SEs). Covariates included in the multivariable models were selected based on clinical relevance and univariate significance (*p* < 0.10), comprising imaging guidance modality (US-PTBD vs. F-PTBD), access side (right vs. left), and etiology of malignant obstruction (metastases vs. cholangiocarcinoma vs. pancreatic cancer). Model fit and multicollinearity were assessed using variance inflation factors (VIFs < 2.5). Statistical analyses were performed using IBM SPSS Statistics version 26.0 (IBM Corp., Armonk, NY, USA).

## 3. Results

### 3.1. Study Population

During the study period, spanning from January 2018 to December 2025, a total of 133 patients (83 males and 50 females; mean age 69 years) underwent percutaneous transhepatic biliary drainage (PTBD) for the management of malignant obstructive jaundice, resulting in a cumulative total of 163 procedures. Among these, 118 procedures were performed under fluoroscopic guidance (F-PTBD), whereas 45 interventions were carried out under ultrasound guidance (US-PTBD). The primary etiologies of malignant biliary obstruction across the cohort were metastases (*n* = 87; 53.4%), cholangiocarcinoma (*n* = 42; 25.8%), and pancreatic cancer (*n* = 34; 20.8%). As detailed in Table 1, a comparative analysis of baseline demographic and clinical characteristics demonstrated that the two guidance cohorts were well-balanced and homogeneous, with no statistically significant differences in mean age (69.2 ± 8.5 vs. 68.6 ± 8.1 years; *p* = 0.677), male sex proportion (62.7% vs. 64.4%; *p* = 0.981), distribution of underlying malignancies (*p* = 0.979), stricture location (*p* = 0.860) or prevalence of baseline comorbidities—including hypertension, diabetes mellitus, cardiovascular disease, and ECOG performance status (all *p* > 0.05).

### 3.2. Procedure Data

Table 2 provides a comprehensive comparison of the procedural characteristics and outcomes of PTBD interventions performed under fluoroscopic and ultrasound guidance. A statistically significant difference was observed in the laterality of access, with F-PTBD procedures predominantly performed on the right side (91.5%) compared to only 31% in the US-PTBD group (*p* < 0.001). Conversely, US-PTBD was more frequently performed on the left side (69%), likely reflecting operator preference and anatomical feasibility under ultrasound guidance. Notably, in five patients, a switch in access side was necessary due to procedural challenges: three patients initially approached from the right side required a shift to the left, whereas two patients initially attempted from the left required a right-sided approach. Significant differences were observed in key procedural metrics. F-PTBD procedures were associated with a significantly higher dose–area product (DAP), with an average of 6.2 ± 1.5 Gy·cm^2^, compared to 4.1 ± 1.9 Gy·cm^2^ in US-PTBD (*p* < 0.001). Similarly, the mean total procedure time was substantially longer in the F-PTBD group (56.6 ± 6 min) compared to the US-PTBD group (42 ± 7 min, *p* < 0.001). Fluoroscopy time was also significantly prolonged in the F-PTBD group (17.4 ± 2.8 min vs. 13 ± 4.1 min, *p* < 0.001).

Despite these differences, the technical success rate between the two approaches was not statistically different, with an 82.2% success rate for F-PTBD and 91.1% for US-PTBD (*p* = 0.158). The main reasons for US-PTBD failure were the inability to pass the guidewire into the biliary system due to a non-anatomically favorable needle position. Early bilirubin reduction at 5–7 days was observed in both groups, with a mean absolute reduction of 6.20 ± 1.30 mg/dL (39.48% ± 19.43%) in Group 1 and 5.80 ± 1.70 mg/dL (42.33% ± 20.70%) in Group 2. No statistically significant difference was observed between groups regarding absolute bilirubin reduction (Welch’s t = 1.414, *p* = 0.162) or relative percentage reduction (Mann–Whitney U = 2452.0, *p* = 0.452). The fluoroscopy-guided group showed a numerically higher complication rate than the ultrasound-guided group; however, this difference did not reach statistical significance (10.2% vs. 6.7%, *p* = 0.489). Specifically, in the US-PTBD group, only minor complications were recorded (i.e., mild pain, nausea, vomiting, and abdominal wall hematoma in the absence of significant bleeding). In contrast, F-PTBD was associated with a greater incidence of major complications, including four cases of significant bleeding (defined as a hemoglobin drop of ≥3 g/dL) and eight cases of post-procedural cholangitis. Additionally, the number of percutaneous punctures through Glisson’s capsule was higher in the F-PTBD group (*p* = 0.043), consistent with increased access difficulty and potentially greater procedural trauma.

### 3.3. Right Access vs. Left Access

Table 3 presents a detailed comparison between right-sided and left-sided PTBD access, highlighting key differences in procedural characteristics, technical success rates, radiation exposure, and complications. Overall, technical success rates were comparable between the two approaches, with 85.2% of right-sided PTBD procedures and 82.9% of left-sided PTBD procedures achieving successful biliary drainage (*p* = 0.721). However, a notable distinction emerged in the choice of imaging guidance. Right-sided PTBD was predominantly performed under fluoroscopic guidance, with 86.8% of procedures relying on fluoroscopy compared to only 13.2% performed with ultrasound guidance. In contrast, left-sided access was primarily ultrasound-guided, accounting for 70.7% of cases, while only 29.3% required fluoroscopic support (*p* < 0.001). Fluoroscopy-dominant right-sided access resulted in substantially higher radiation exposure, as reflected by a significantly elevated dose–area product (DAP). The mean DAP for right-sided PTBD was 6 ± 1.1 Gy·cm^2^, compared to 4.6 ± 3 Gy·cm^2^ for left-sided access (*p* < 0.001). Additionally, right-sided access was associated with significantly longer procedure and fluoroscopy times. The mean total procedure time for right-sided PTBD was 55.4 ± 5.9 min, whereas left-sided procedures were completed in a shorter average duration of 44 ± 11.2 min (*p* < 0.001). Fluoroscopy time followed a similar trend, with right-sided interventions requiring a mean of 17 ± 2.2 min compared to 13.8 ± 5.9 min in left-sided cases (*p* < 0.001). In terms of procedural difficulty, the number of percutaneous punctures required to achieve successful biliary access was slightly higher in the right-sided PTBD group, with an average of 1.77 ± 0.18 punctures compared to 1.29 ± 0.19 punctures for left-sided access (*p* = 0.049). Finally, the overall complication rates did not differ significantly between the two approaches, with 10.6% of right-sided PTBD cases experiencing complications compared to 4.9% in the left-sided group (*p* = 0.268). Although the left-sided approach demonstrated a trend toward fewer complications, this difference was not statistically significant.

Table 4 and Table 5 present a comparison of fluoroscopy-guided (F-PTBD) and ultrasound-guided (US-PTBD) procedures for both right-sided and left-sided PTBD, highlighting differences in technical success rates, radiation exposure, procedure duration, and complications.

For right-sided PTBD (Table 4), technical success rates were similar between the two guidance methods, with F-PTBD achieving successful biliary drainage in 84.9% of cases and US-PTBD in 87.5% of cases (*p* = 0.785). However, F-PTBD procedures resulted in significantly higher radiation exposure, with a mean dose–area product (DAP) of 6.9 ± 1.1 Gy·cm^2^ compared to 5.89 ± 1.06 Gy·cm^2^ in the US-PTBD group (*p* = 0.003). Additionally, F-PTBD was associated with longer procedure times (mean 59.5 ± 5.2 min vs. 46.6 ± 3.1 min, *p* < 0.001) and longer fluoroscopy times (mean 20.3 ± 5.2 min vs. 16.7 ± 2.1 min, *p* = 0.003). The number of percutaneous punctures required to gain biliary access was also higher in the F-PTBD group, with a mean of 1.91 ± 0.34 punctures compared to 1.24 ± 0.34 punctures in the US-PTBD group (*p* = 0.041). Complication rates did not show a statistically significant difference between the two methods, with 10.4% of F-PTBD cases and 12.5% of US-PTBD cases experiencing complications (*p* = 0.797).

For left-sided PTBD (Table 5), a significant difference in technical success rates was observed between the two guidance methods. US-PTBD had a higher success rate of 93.1% compared to 58.3% for F-PTBD (*p* = 0.007). Radiation exposure was also markedly lower in the US-PTBD group, with a mean DAP of 2.9 ± 0.3 Gy·cm^2^ versus 9.9 ± 0.9 Gy·cm^2^ in the F-PTBD group (*p* < 0.001). Furthermore, US-PTBD procedures were completed in a shorter time, with a mean procedure duration of 39.9 ± 7.2 min compared to 56.8 ± 12.2 min for F-PTBD (*p* < 0.001). Fluoroscopy time was also significantly lower in US-PTBD cases (10.5 ± 1 min vs. 24 ± 1 min, *p* < 0.001). The number of percutaneous punctures was lower in the US-PTBD group, with a mean of 1.15 ± 0.11 punctures compared to 1.63 ± 0.21 punctures in the F-PTBD group (*p* = 0.049). Complication rates were low in both groups and did not differ significantly, with 8.3% of F-PTBD cases and 3.4% of US-PTBD cases experiencing complications (*p* = 0.508).

These data demonstrate that fluoroscopy-guided procedures were associated with higher radiation exposure, longer procedure duration, and a greater number of percutaneous punctures compared to ultrasound-guided procedures, regardless of access side. The technical success rate was higher in the US-PTBD group for left-sided access, while complication rates did not differ significantly between the two techniques.

In the multivariable regression models adjusting for access side (right vs. left) and tumor etiology, ultrasound guidance (US-PTBD) remained an independent predictor of significantly lower radiation exposure (DAP: adjusted beta = −1.82, 95% CI: −2.41 to −1.23, *p* < 0.001), shorter total procedure time (adjusted beta = −11.84 min, 95% CI: −14.32 to −9.36, *p* < 0.001), and fewer Glisson’s capsule punctures (adjusted beta = −0.48, 95% CI: −0.72 to −0.24, *p* = 0.001). Access side was also an independent determinant of procedural duration and dose, with left-sided access associated with reduced DAP independently of guidance modality. Regarding safety and technical performance, US-PTBD showed a non-significant trend toward higher technical success (aOR = 1.85, 95% CI: 0.58 to 5.91, *p* = 0.298) and lower overall complications (aOR = 0.54, 95% CI: 0.14 to 2.08, *p* = 0.372) after adjusting for confounding baseline and procedural factors (Table 6).

## 4. Discussion

The medical innovations of recent years have undoubtedly expanded treatment options for patients with obstructive jaundice, yet they have not rendered percutaneous transhepatic biliary drainage (PTBD) obsolete in clinical practice [31]. PTBD continues to serve as a vital alternative to endoscopic retrograde cholangiopancreatography (ERCP) when ERCP is unsuccessful or infeasible, maintaining its utility particularly among patients with altered gastrointestinal anatomy, such as biliodigestive anastomoses following major surgery [32,33,34]. Although fluoroscopy is commonly used for cholangiography and device manipulation during PTBD, the initial duct puncture step can be performed under ultrasound or fluoroscopy and may meaningfully affect capsule passes, fluoroscopy time, and radiation exposure. This study isolates that procedural component and further explores right- vs. left-sided access, where comparative data are limited [10,11]. Our findings emphasize the advantages of ultrasound guidance, particularly in reducing radiation exposure and procedural time, with a comparable technical success rate to fluoroscopy. These insights are especially valuable given the scarcity of direct comparative studies on US-PTBD and F-PTBD.

Our study identified a trend toward a higher complication rate in the F-PTBD group (10.2%) compared to the US-PTBD group (6.7%), albeit without statistical significance (*p* = 0.489). A type II error shouldn’t be excluded, given the limited sample size of our population [35]. Notably, major complications, including post-procedural cholangitis and significant bleeding, occurred only in the F-PTBD cohort. This observation aligns with findings from Kozlov et al. [36], who reported a 17.6% complication rate in F-PTBD compared to 5.9% in US-PTBD. Additionally, Giurazza et al. (2019) highlighted the safety of US-PTBD, where minor complications were the predominant adverse events [37]. Similarly, no difference regarding the overall interventional complication rate (10.6% vs. 9.1%) was observed by Nennstiel et al., but major complications were only encountered in F-PTBDs [38]. The lower number of Glisson capsule punctures in US-PTBD compared to F-PTBD (1.23 ± 0.21 vs. 1.81 ± 0.19, *p* = 0.043) supports the hypothesis that real-time vascular and biliary anatomy visualization with ultrasound reduces procedure-related trauma. The significant absence of major bleeding events in the US-PTBD group underscores its safety profile, particularly in patients with coagulopathy or high-risk anatomy, a critical advantage over fluoroscopy-guided techniques. However, dedicated studies are needed before recommending a specific biliary duct access technique on the basis of coagulopathy status. The numerically higher complication rate in the fluoroscopy-guided group may also reflect the predominance of right-sided intercostal access, which may entail pleural and vascular risks distinct from those of epigastric left-sided access, rather than the imaging modality alone. A systematic review of the literature also showed that US-PTBD has lower median rates of severe early complications (0% versus 8%) and procedural death (0% versus 1%) than F-PTBD [39]. Conversely, in a recent systematic review and meta-analysis, the US group didn’t show an improved bleeding rate in PTBD [39]. Finally, future multicenter studies with larger populations may better demonstrate a possible advantage of the US over fluoroscopy in terms of overall complication and major complication rates. Pulappadi et al. (2021) discussed the diagnosis and management of hemorrhagic complications associated with PTBD [40]. The incidence of hemorrhage after percutaneous biliary interventions varies from 1.1% to 9.9%. Overall complication rates of PTBD are similar to endoscopic biliary drainage, ranging from 12.9% to 67%. Risk factors for hemorrhagic complications include advanced age (≥73 years), chronic kidney disease, cirrhosis, use of large bore puncture needles (18G), central duct puncture, minimally dilated or non-dilated bile ducts, multiple passes, multiple catheter placements, ascites, deranged coagulation parameters, and ongoing use of antithrombotic agents. Interestingly, our study recorded a very low rate of bleeding, thus highlighting the pivotal role of proper technique and imaging guidance to minimize the hemorrhage risk.

Regarding the etiology of biliary obstruction, metastatic disease was the most frequently observed cause, accounting for 87 cases (53%) within the entire cohort. Cholangiocarcinoma was the second most prevalent malignancy, representing 42 cases (26%), while pancreatic cancer was responsible for 34 cases (21%). These findings are consistent with previous literature, where metastatic disease and primary hepatobiliary malignancies are recognized as leading causes of malignant biliary obstruction requiring percutaneous drainage [41]. Given the high proportion of patients with advanced-stage malignancy, a significant number of cases involved complex biliary anatomy and/or poor clinical status, underscoring the importance of selecting the most appropriate imaging modality to optimize procedural success while minimizing complications.

US-PTBD demonstrated markedly shorter procedural and fluoroscopy times than F-PTBD, with an average procedural time of 42 ± 7 min versus 56.6 ± 6 min (*p* < 0.001) which may be attributed to the additional steps required for fluoroscopic navigation and contrast-based biliary opacification. In terms of radiation exposure, US-PTBD offers a significant advantage, with a mean dose–area product (DAP) of 4.1 ± 1.9 Gy·cm^2^ compared to 6.2 ± 1.5 Gy·cm^2^ for F-PTBD (*p* < 0.001). Our findings are consistent with those observed by Park et al. on 50 PTBD procedures with US guidance (mean total DAP = 5.2 ± 3.8 Gy·cm^2^) [42]. A study by Schmitz et al. reported a similar radiation exposure [43]. This reduction has critical implications for patient safety, particularly in repeated procedures, and for healthcare providers routinely exposed to ionizing radiation [44]. Real-time ultrasound guidance enhances procedural accuracy, facilitating precise needle placement, thus minimizing procedural time and radiation exposure, which is especially advantageous in anatomically complex cases. However, this finding must be interpreted cautiously because the ultrasound-guided group was predominantly left-sided, whereas the fluoroscopy-guided group was predominantly right-sided. Since left-sided punctures are often technically more direct and less constrained by intercostal access, part of the observed difference may be attributable to access-side distribution rather than guidance modality alone. A major strength of the present study is the implementation of multivariable regression analysis to adjust for potential confounding factors, particularly access side and obstruction etiology. Because left-sided access was predominantly performed under ultrasound guidance in our cohort (69% vs. 8.5% in the fluoroscopy group), univariate reductions in radiation exposure and procedural time could have been partially driven by anatomical access selection. However, our multivariable analysis demonstrated that ultrasound guidance independently predicts lower Dose–Area Product (beta = −1.82 Gy cm^2^, *p* < 0.001), shorter procedural duration (beta = −11.84 min, *p* < 0.001), and fewer capsule punctures (beta = −0.48, *p* = 0.001), even after controlling for access side. These findings confirm that the radiation-sparing and procedural efficiency benefits of US-PTBD are intrinsically linked to real-time sonographic visualization of intrahepatic ducts during initial access. Furthermore, after multivariable adjustment, complication rates remained statistically comparable between modalities, reinforcing that US-PTBD achieves these operational advantages without compromising patient safety or technical success.

The technical success rates for US-PTBD (91.1%) and F-PTBD (82.2%) in our study did not differ significantly (*p* = 0.158). This similarity suggests that both access routes can be effectively utilized depending on individual patient anatomy and operator preference. Interestingly, US-PTBD demonstrated a higher technical success rate in left-sided access (93.1% vs. 58.3%, *p* = 0.007), suggesting that ultrasound guidance may be particularly advantageous in anatomically complex cases. These findings are consistent with Nennstiel et al. [38], who reported a similar overall technical success rate of 85–90% for both methods, albeit with some variability depending on the access side. Wagner et al. observed that US-PTBD was technically successful in 94% of attempts with a mean of 2.2 needle passes [16]. Importantly, we also assessed early biochemical response through bilirubin reduction at 5–7 days, which provides a clinically meaningful complement to technical success. Both cohorts demonstrated a robust and comparable early functional recovery, with mean absolute bilirubin reductions of 6.20 ± 1.30 mg/dL (39.48% ± 19.43%) in Group 1 and 5.80 ± 1.70 mg/dL (42.33% ± 20.70%) in Group 2, showing no statistically significant differences in either absolute (*p* = 0.162) or relative percentage terms (*p* = 0.452). A rapid decline in serum bilirubin within the first post-procedural week represents a key surrogate marker for effective biliary decompression, which is essential to restore hepatic function, reduce the incidence of post-procedural cholangitis, and prevent delays in initiating adjuvant systemic chemotherapy. These findings align with previous literature evaluating biliary drainage outcomes; for example, Sharaiha et al. (2016) and Zhang et al. (2014) similarly demonstrated that successful biliary decompression typically yields an early relative bilirubin clearance of 20% to 50% within 7 days, with no significant divergence between different drainage modalities [45,46]. Taken together, our results indicate that both approaches provide equivalent early biochemical efficacy, confirming that the choice of procedure can be safely tailored to patient anatomy and operator expertise without compromising short-term functional recovery.

In recent years, endoscopic ultrasound-guided biliary drainage (EUS-BD) has emerged as an increasingly recognized alternative to percutaneous biliary drainage, particularly in patients with malignant biliary obstruction after failed or unsuccessful ERCP. EUS-BD may provide effective biliary decompression while avoiding external drainage and reducing the need for percutaneous access [46]. However, its application remains dependent on local expertise, equipment availability, patient anatomy, and the feasibility of the procedure. Therefore, despite the increasing role of EUS-BD in contemporary biliary drainage algorithms, PTBD remains an important and widely applicable option, particularly when EUS-BD is unavailable, technically infeasible, or not routinely performed. In this context, comparison of different image-guidance strategies for PTBD remains clinically relevant.

While our findings highlight the potential benefits of US-PTBD, this study has several limitations. First, its retrospective design introduces potential selection biases, particularly in access route and guidance modality, which were determined by operator preference. This non-random allocation reflects real-world clinical practice but also introduces indication bias, as more technically favorable cases may have been preferentially selected for ultrasound-guided access. Second, the relatively small sample size of US-PTBD procedures, particularly for right-sided access, may limit the generalizability of our findings. Subgroup analyses by access side should also be interpreted with caution because some comparator groups were small, particularly left-sided F-PTBD. As a result, these analyses may be underpowered and vulnerable to unstable estimates, even when effect sizes appear large. Third, long-term outcomes, such as patient survival, were not evaluated, precluding a comprehensive assessment of clinical efficacy. Finally, operator expertise with ultrasound-guided procedures may have influenced the outcomes, as proficiency in this technique varies significantly among interventional radiologists. Procedures were performed by two experienced operators (10 and 30 years), which may limit generalizability to lower-volume and less experienced settings. Addressing these limitations through multicenter studies involving a larger, diverse patient population could offer a more comprehensive validation of observed trends. Moreover, standardized protocols across different facilities would be beneficial in mitigating variability and ensuring consistency in outcomes.

## 5. Conclusions

US-PTBD was associated with lower radiation exposure and shorter procedure duration compared with F-PTBD in this retrospective cohort. However, these findings should be interpreted with caution given the marked imbalance in access side between the two groups, with US-PTBD being predominantly performed through left-sided access and F-PTBD through right-sided access. In addition, the retrospective, non-randomized study design and the potential for operator-related and indication bias may have influenced the observed differences. Therefore, while US-PTBD may offer relevant procedural advantages, these findings should not be interpreted as definitive evidence of superiority over F-PTBD. Prospective studies with standardized access strategies and adjustment for relevant procedural and patient-related factors are warranted to further clarify the comparative role of ultrasound- and fluoroscopy-guided PTBD.

## Figures and Tables

**Table 1:** Baseline demographic and clinical characteristics of the study population according to guidance modality.

Characteristics	F-PTDB (*n* = 118)	US-PTDB (*n* = 45)	*p*-Value
Number of Procedures, n (%)	118 (72.4%)	45 (27.6%)	NA
Sex			0.981
- *Male*	74 (62.7%)	29 (64.4%)	
- *Female*	44 (37.3%)	16 (35.6%)	
Age (years), Mean ± SD	69.2 ± 8.5	68.6 ± 8.1	0.677
Etiology of Malignant Obstruction, n (%)			0.979
- *Metastases*	63 (53.4%)	24 (53.3%)	
- *Cholangiocarcinoma*	30 (25.4%)	12 (26.7%)	
- *Pancreatic cancer*	25 (21.2%)	9 (20.0%)	
Comorbidities & Health Status, n (%)			
- *Hypertension*	67 (56.8%)	25 (55.6%)	1.000
- *Diabetes mellitus*	30 (25.4%)	11 (24.4%)	1.000
- *Cardiovascular disease*	26 (22.0%)	9 (20.0%)	0.945
*ECOG Performance Status ≥ 2*	38 (32.2%)	14 (31.1%)	1.000
Stricture Location, n (%)			0.860
- *Hilar/proximal*	68 (57.6%)	25 (55.6%)	
- *Distal*	50 (42.4%)	20 (44.4%)	

**Table 2:** Characteristics of the PTBD interventions, according to the type of guidance.

Characteristics	F-PTDB (n = 118)	US-PTDB (n = 45)	*p*-Value
Technical success, n	97 (82.2%)	41 (91.1%)	0.158
5–7 days Bilirubin reduction (mg/dL), absolute change	6.2 ± 1.3	5.8 ± 1.7	0.162
5–7 days Bilirubin reduction (mg/dL), percentage	39.48 ± 19.43%	42.33 ± 20.70%	0.452
Side			<0.001
- *Right*	108 (91.5%)	14 (31%)	
- *Left*	10 (8.5%)	31 (69%)	
Dose–area product (DAP, Gy·cm^2^), mean	6.2 ± 1.5	4.1 ± 1.9	<0.001
Procedure time (min), mean	56.6 ± 6	42 ± 7	<0.001
Fluoroscopy time (min), mean	17.4 ± 2.8	13 ± 4.1	<0.001
Punctures, n	1.81 ± 0.19	1.23 ± 0.21	0.043
Complications, n	12 (10.2%)	3 (6.7%)	0.489

**Table 3:** Right-access versus left-access data.

Characteristics	Right Access (n = 122)	Left Access (n = 41)	*p*-Value
Technical success, n	104 (85.2%)	34 (82.9%)	0.721
Guide (Fluoroscopy/US)	106 (86.8%)/16 (13.2%)	12 (29.3%)/29 (70.7%)	<0.001
Dose–area product (DAP, Gy·cm^2^), mean	6 ± 1.1	4.6 ± 3	<0.001
Procedure time (min), mean	55.4 ± 5.9	44 ± 11.2	<0.001
Fluoroscopy time (min), mean	17 ± 2.2	13.8 ± 5.9	<0.001
Punctures, n	1.77 ± 0.18	1.29 ± 0.19	0.049
Complications, n	13 (10.6%)	2 (4.9%)	0.268

**Table 4:** Right-access data, according to the type of guidance.

Characteristics	Right Access F-PTDB (n = 106)	Right Access US-PTDB (16)	*p*-Value
Technical success, n	90 (84.9%)	14 (87.5%)	0.785
Dose–area product (DAP, Gy·cm^2^), mean	6.9 ± 1.1	5.89 ± 1.06	0.003
Procedure time (min), mean	59.5 ± 5.2	46.6 ± 3.1	<0.001
Fluoroscopy time (min), mean	20.3 ± 5.2	16.7 ± 2.1	0.003
Punctures, n	1.91 ± 0.34	1.24 ± 0.34	0.041
Complications, n	11 (10.4%)	2 (12.5%)	0.797

**Table 5:** Left-access data, according to the type of guidance.

Characteristics	Left Access F-PTDB (n = 12)	Left Access US-PTDB (n = 29)	*p*-Value
Technical success, n	7 (58.3%)	27 (93.1%)	0.007
Dose–area product (DAP, Gy·cm^2^), mean	9.9 ± 0.9	2.9 ± 0.3	<0.001
Procedure time (min), mean	56.8 ± 12.2	39.9 ± 7.2	<0.001
Fluoroscopy time (min), mean	24 ± 1	10.5 ± 1	<0.001
Punctures, n	1.63 ± 0.21	1.15 ± 0.11	0.049
Complications, n	1 (8.3%)	1 (3.4%)	0.508

**Table 6:** Multivariable Linear and Logistic Regression Analysis for Procedural Outcomes and Complications.

Outcome Measure	Variable/Model Covariates	Adjusted Effect (Beta or aOR)	95% Confidence Interval	*p*-Value
Dose–Area Product (DAP, Gy·cm^2^)	Guidance (US vs. Fluoroscopy)	Beta = −1.82	−2.41 to −1.23	<0.001
	Access Side (Left vs. Right)	Beta = −0.85	−1.48 to −0.22	0.009
	Tumor Etiology (Cholangiocarcinoma)	Beta = +0.12	−0.45 to +0.69	0.680
Procedure Time (min)	Guidance (US vs. Fluoroscopy)	Beta = −11.84	−14.32 to −9.36	<0.001
	Access Side (Left vs. Right)	Beta = −4.12	−6.85 to −1.39	0.003
Punctures (n)	Guidance (US vs. Fluoroscopy)	Beta = −0.48	−0.72 to −0.24	0.001
	Access Side (Left vs. Right)	Beta = −0.21	−0.48 to +0.06	0.128
Technical Success	Guidance (US vs. Fluoroscopy)	aOR = 1.85	0.58 to 5.91	0.298
	Access Side (Left vs. Right)	aOR = 0.92	0.31 to 2.74	0.881
Complications	Guidance (US vs. Fluoroscopy)	aOR = 0.54	0.14 to 2.08	0.372
	Access Side (Left vs. Right)	aOR = 0.51	0.10 to 2.58	0.418

Note: beta represents unstandardized linear regression coefficients for continuous outcomes; aOR represents adjusted odds ratios from multivariable logistic regression for binary outcomes.

## Data Availability

The data presented in this study are available on request from the corresponding author. The data are not publicly available due to privacy issues.

## Abbreviations

The following abbreviations are used in this manuscript:

DAP	Dose–area product
ERCP	Endoscopic retrograde cholangiopancreatography
EUS	Endoscopic ultrasound
F-PTBD	Fluoroscopy-guided percutaneous trans-hepatic biliary drainage
PTBD	Percutaneous trans-hepatic biliary drainage
PTC	Percutaneous transhepatic cholangiography
US-PTBD	Ultrasound-guided percutaneous trans-hepatic biliary drainage
